# Supplementary material for: Orbital- and millennial-scale Asian winter monsoon variability across the Pliocene–Pleistocene glacial intensification
Source: Nat Commun. 2024 Apr 19;15:3364. doi: 10.1038/s41467-024-47274-9 (PMC11031568; doi:10.1038/s41467-024-47274-9)
Supplement: Supplementary file 1 — Supplementary Information [file 41467_2024_47274_MOESM1_ESM.pdf]

Supplementary Information

Orbital- and millennial-scale Asian winter monsoon variability across the Pliocene–Pleistocene glacial intensification

Ao et al.

Supplementary Note 1: Asian summer monsoon variability across the Pliocene–Pleistocene glacial intensification

The Asian summer monsoon (ASM) had a more complex orbital-scale variability than the Asian winter monsoon (AWM) during the late Pliocene–early Pleistocene. Magnetic susceptibility (χ) records from the Chinese Loess Plateau (CLP) are often used to infer ASM variability, although the exact relationship between χ and pedogenic intensity remains debated for the red clay sequence[1-3]. Coeval with AWM strengthening, several CLP χ records suggest ASM weakening across the intensification of northern hemisphere glaciation (iNHG)[4,5] (Supplementary Fig. 11g–j). Those χ records suggest that the ASM had a distinct orbital variability largely corresponding to glacial-interglacial global climate cycles after 2.7 Ma, while the parallel AWM records appear to be anti-correlated. However, such distinct orbital-scale variability is largely subdued or even absent in those records before 2.7 Ma, possibly caused by χ smoothing in red clay sequences associated with low dust accumulation rates under warmer, higher CO_2, and less glaciated Pliocene conditions[6,7]. Gamma-ray sediment borehole logs from Taiwan[8] and a hematite to goethite ratio (Hm/Gt) record from the South China Sea[9] suggest that orbital-scale ASM variability largely matched global glacial-interglacial cycles from the late Pliocene to early Pleistocene (Supplementary Fig. 11k–l) but is anti-correlated to coeval AWM variability as recorded by the Chongxin grain size record. Nevertheless, a K/Al record from the South China Sea[10] and fluvial-lacustrine Rb/Sr records from the Sanmenxia Basin (central China)[11], Yuanmou Basin (southwestern China)[12], and Qaidam Basin (northwestern China)[13] suggest regionally variable orbital ASM features that differ from the late Pliocene–early Pleistocene glacial-interglacial pacing of global climate and AWM (Supplementary Fig. 11m–p). Therefore, orbital ASM variability and its underlying dynamics continue to be debated across the iNHG. More precisely dated ASM records with more distinct orbital expression are required to resolve orbital-scale ASM variability in the future and in the context of orbital-scale AWM variability.

Supplementary Figures

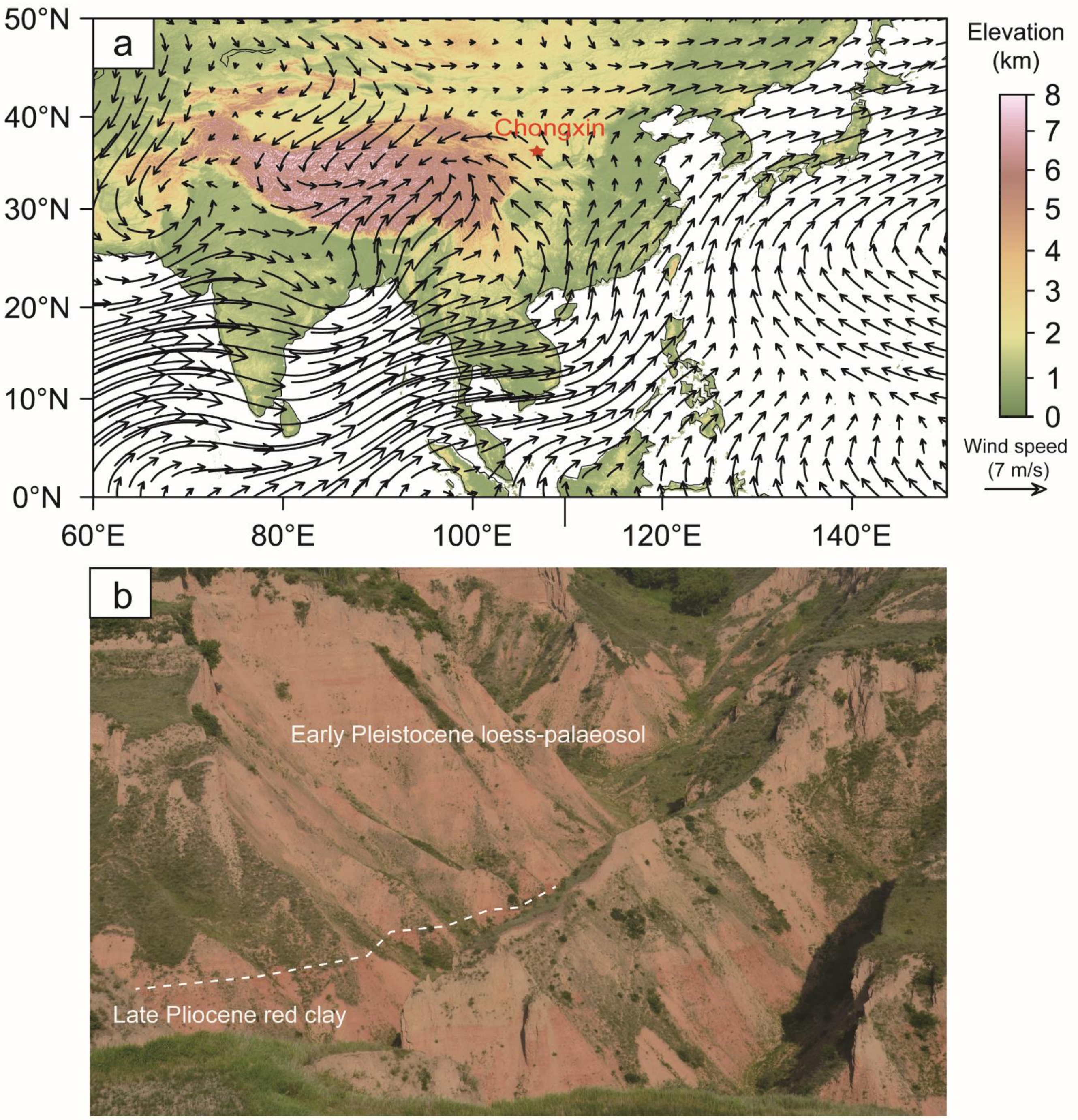

Supplementary Fig. 1. Boreal summer atmospheric circulation and field photograph. (**a**) Topographic map of southern Asia with boreal summer monsoon winds (850 hPa) based on the National Centers for Environmental Prediction/Department of Energy (NCEP/DOE) Reanalysis 2 (NCEP R2) between 1979 and 2020. We created this map with ArcGIS (version 10.7) and Adobe Illustrator 2020 software. (**b**) Field photograph (taken by H. Ao) of late Pliocene red clay and early Pleistocene loess-palaeosol layers (both with horizontal bedding; interpreted Pliocene-Pleistocene boundary in the midground is indicated with a white dashed line; uninterpreted boundary in foreground) from the Chongxin section, central Chinese Loess Plateau.

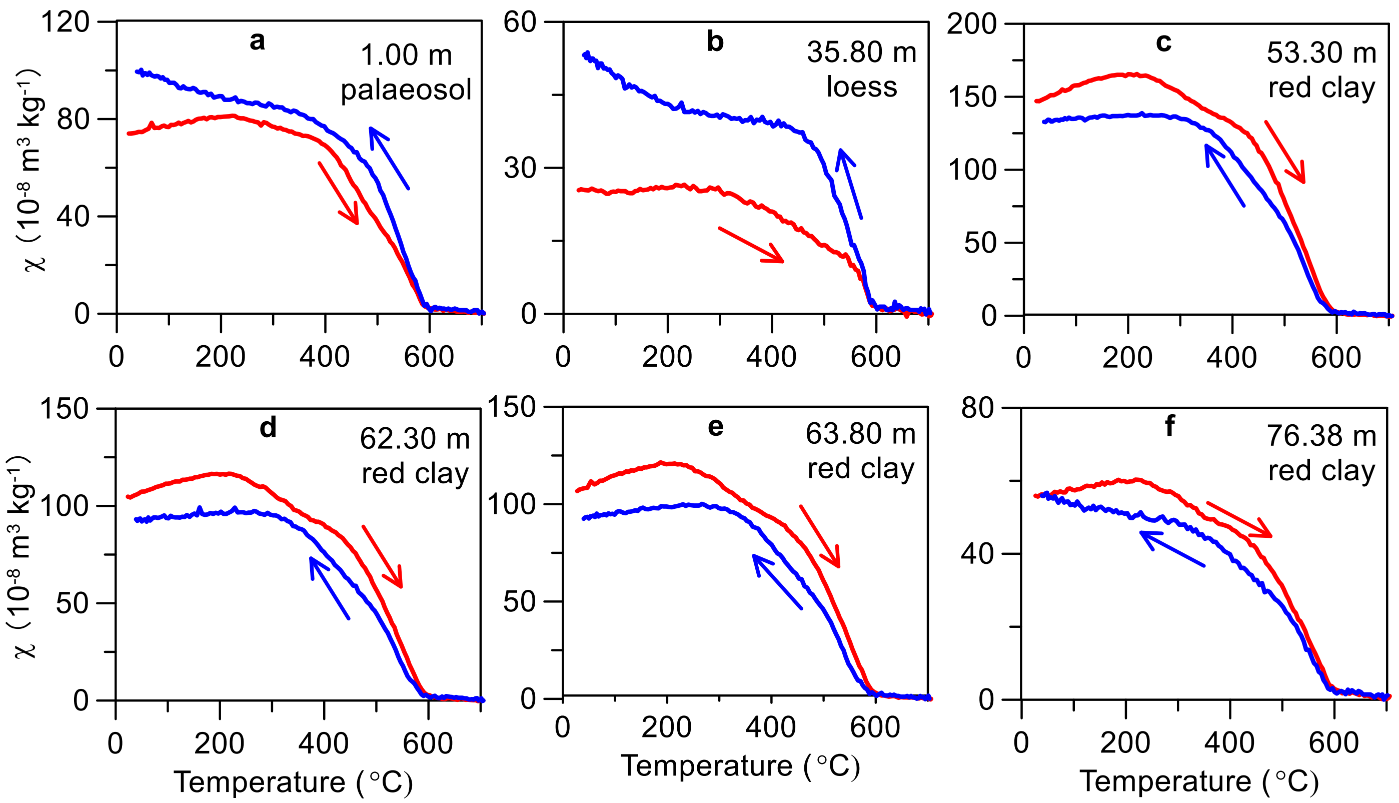

Supplementary Fig. 2. Temperature-dependent magnetic susceptibility (χ–T) curves for selected samples from the Chongxin section. Samples from (**a**) Pleistocene palaeosol at 1 m depth, (**b**) Pleistocene loess at 35.8 m depth, and red clay at (**c**) 53.3 m, (**d**) 62.3 m, (**e**) 63.8 m, and (**f**) 76.38 m depths. Red and blue lines represent heating and cooling curves, respectively. Heating and cooling trajectories (in an argon atmosphere) are indicated by red and blue arrows, respectively.

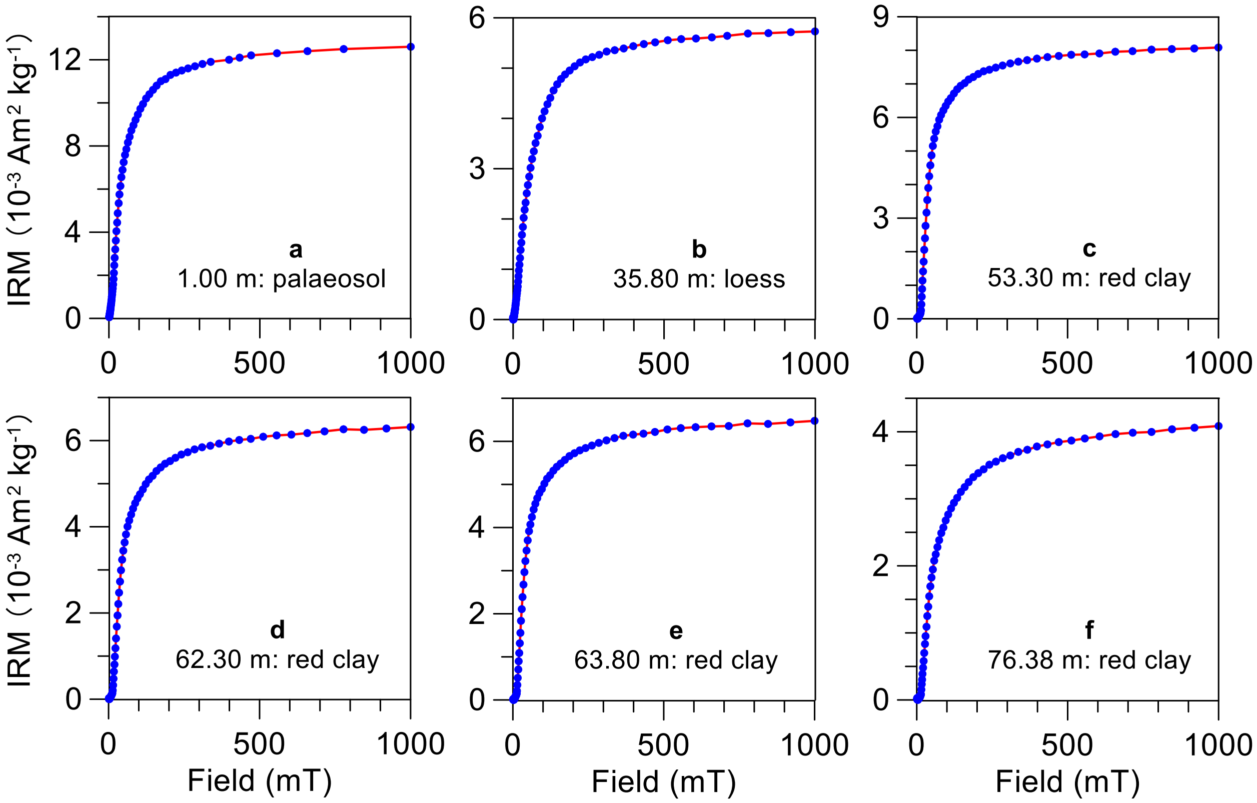

Supplementary Fig. 3. Isothermal remanent magnetization (IRM) acquisition curves for selected samples from the Chongxin section. Samples from (**a**) Pleistocene palaeosol at 1 m depth, (**b**) Pleistocene loess at 35.8 m depth, and red clay at (**c**) 53.3 m, (**d**) 62.3 m, (**e**) 63.8 m, and (**f**) 76.38 m depths.

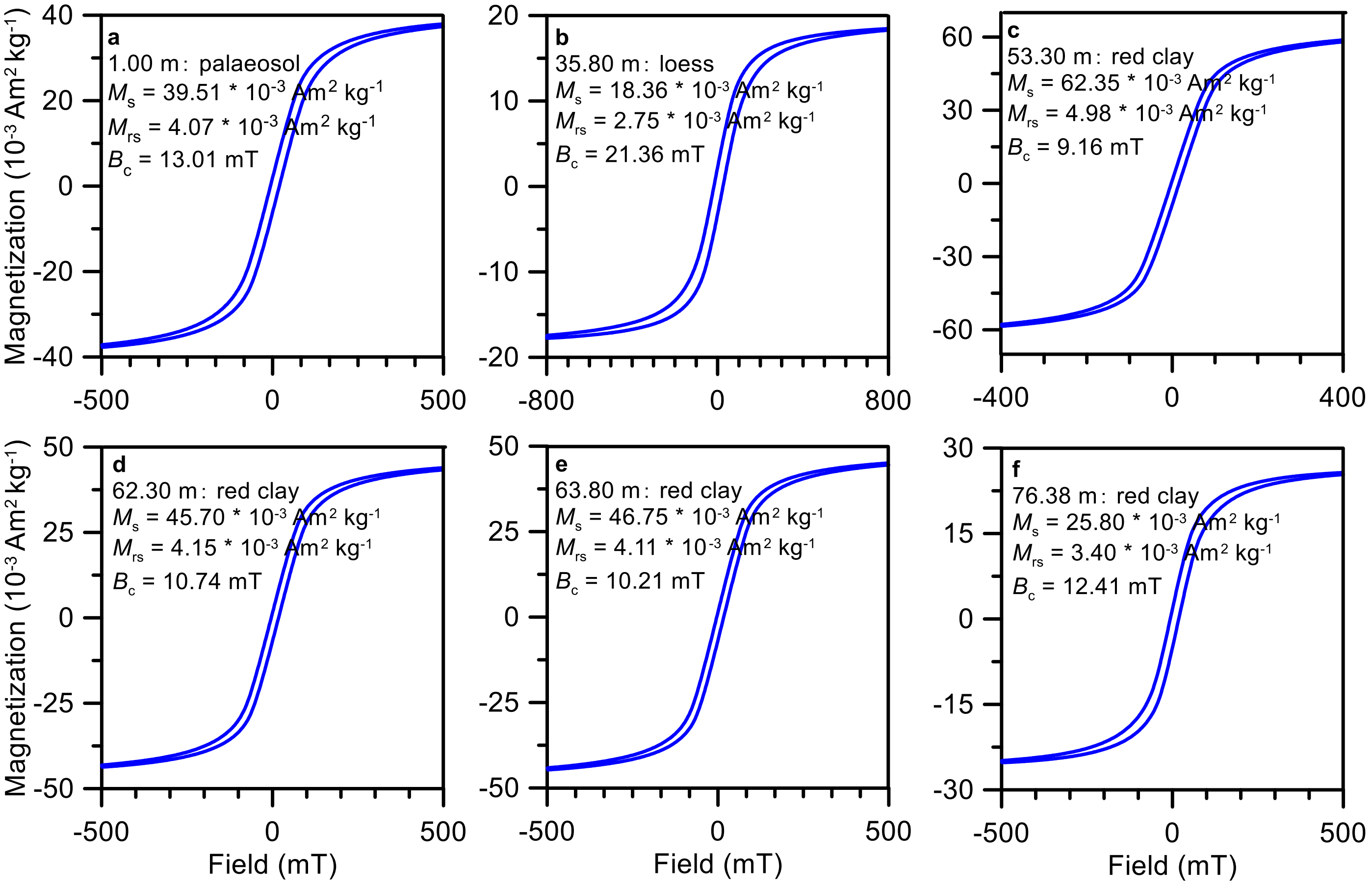

Supplementary Fig. 4. Hysteresis loops for selected samples from the Chongxin section after high-field slope correction. Samples from (**a**) Pleistocene palaeosol at 1 m depth, (**b**) Pleistocene loess at 35.8 m depth, and red clay at (**c**) 53.3 m, (**d**) 62.3 m, (**e**) 63.8 m, and (**f**) 76.38 m depths. Samples were measured to ± 1 T or ± 1.5 T. Central portions of hysteresis loops are shown to better visualize their subtly different behaviour. M_s: saturation magnetization; M_{rs}: saturation remanent magnetization; B_c: coercive force.

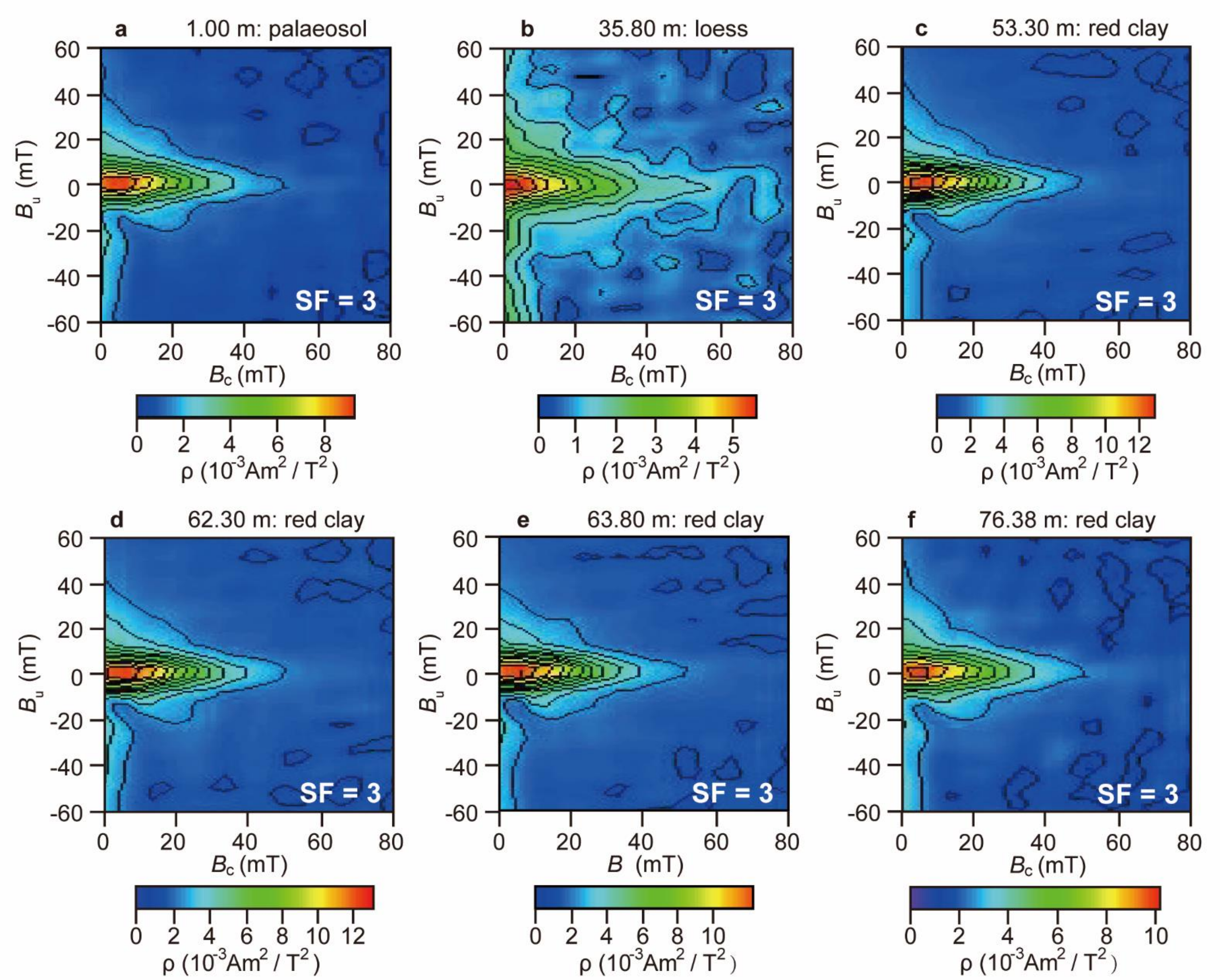

Supplementary Fig. 5. First-order reversal curve (FORC) diagrams for selected samples from the Chongxin section. Samples from (**a**) Pleistocene palaeosol at 1 m depth, (**b**) Pleistocene loess at 35.8 m depth, and red clay at (**c**) 53.3 m, (**d**) 62.3 m, (**e**) 63.8 m, and (**f**) 76.38 m depths. Red colours represent higher FORC distribution densities (ρ). SF: smoothing factor. $B_c = (B_A - B_N)/2$ and $B_u = \pm(B_A + B_N)/2$, where B_N is the vortex nucleation field and B_A is the annihilation field.

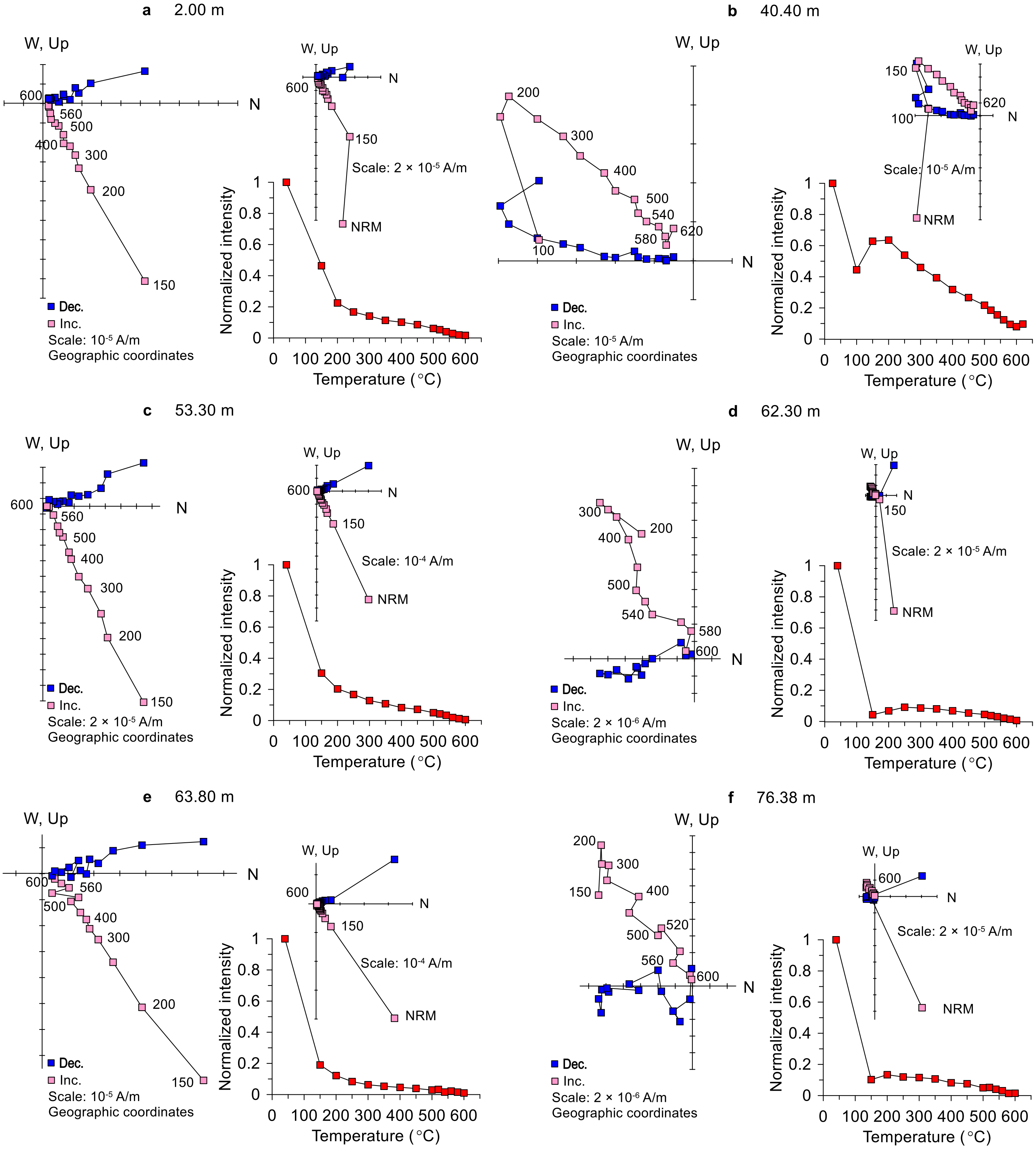

Supplementary Fig. 6. Vector end-point projections and intensity changes of the natural remanent magnetization (NRM) during thermal demagnetization of selected samples from the Chongxin section. Samples from (**a**) Pleistocene palaeosol at 2 m depth, (**b**) Pleistocene loess at 40.4 m depth, and Pliocene red clay at (**c**) 53.3 m, (**d**) 62.3 m, (**e**) 63.8 m, and (**f**) 76.38 m depths. Blue (pink) rectangles represent projections onto the horizontal (vertical) plane[14], with thermal treatment steps indicated in °C next to pink rectangles; red rectangles represent normalized intensity for gradual thermal demagnetization.

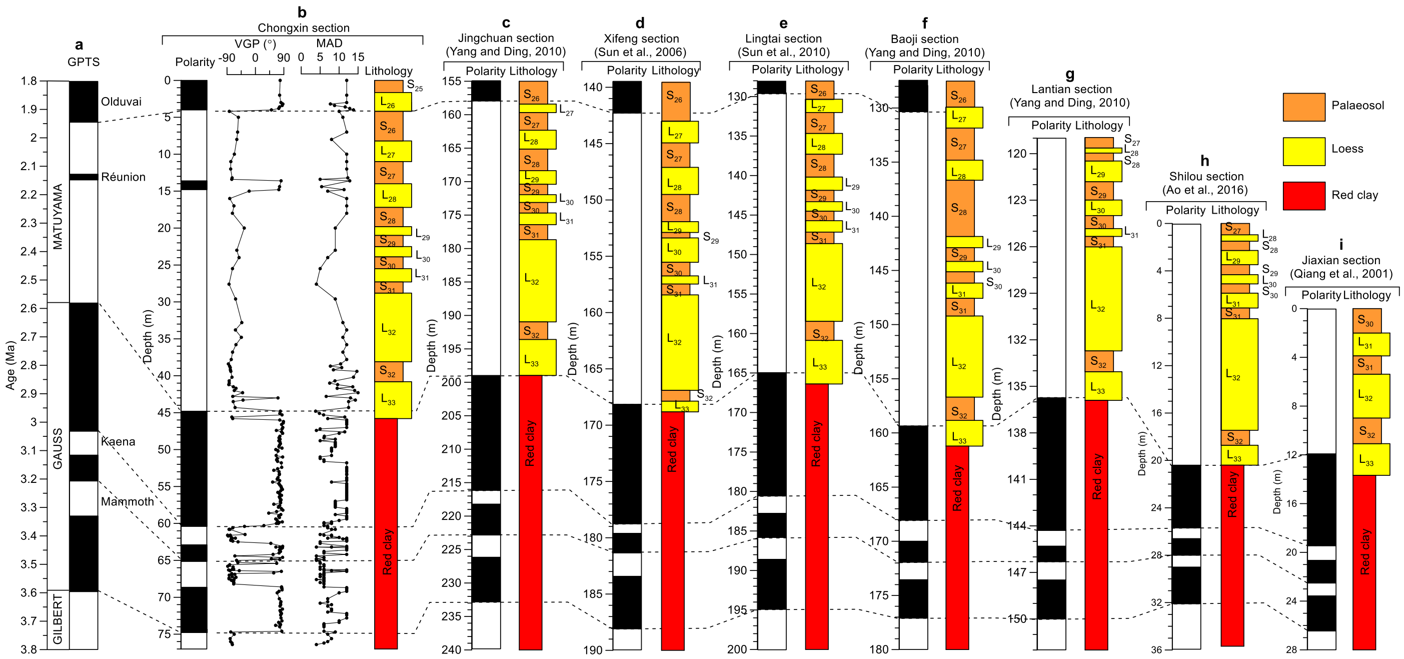

Supplementary Fig. 7. Regional lithostratigraphic and magnetostratigraphic comparison among late Pliocene red clay to early Pleistocene loess-palaeosol sections across the Chinese Loess Plateau. (**a**) geomagnetic polarity timescale (GPTS)[15]. (**b**) Recorded polarity zones with virtual geomagnetic pole (VGP) latitude, maximum angular deviation (MAD) for the principal component analysis fit used to determine the ChRM, and lithology for the Chongxin section. Magnetostratigraphy and lithology for the (**c**) Jingchuan[16], (**d**) Xifeng[5], (**e**) Lingtai[3], (**f**) Baoji[16], (**g**) Lantian[4], (**h**) Shilou[1], and (**i**) Jiaxian[17] sections.

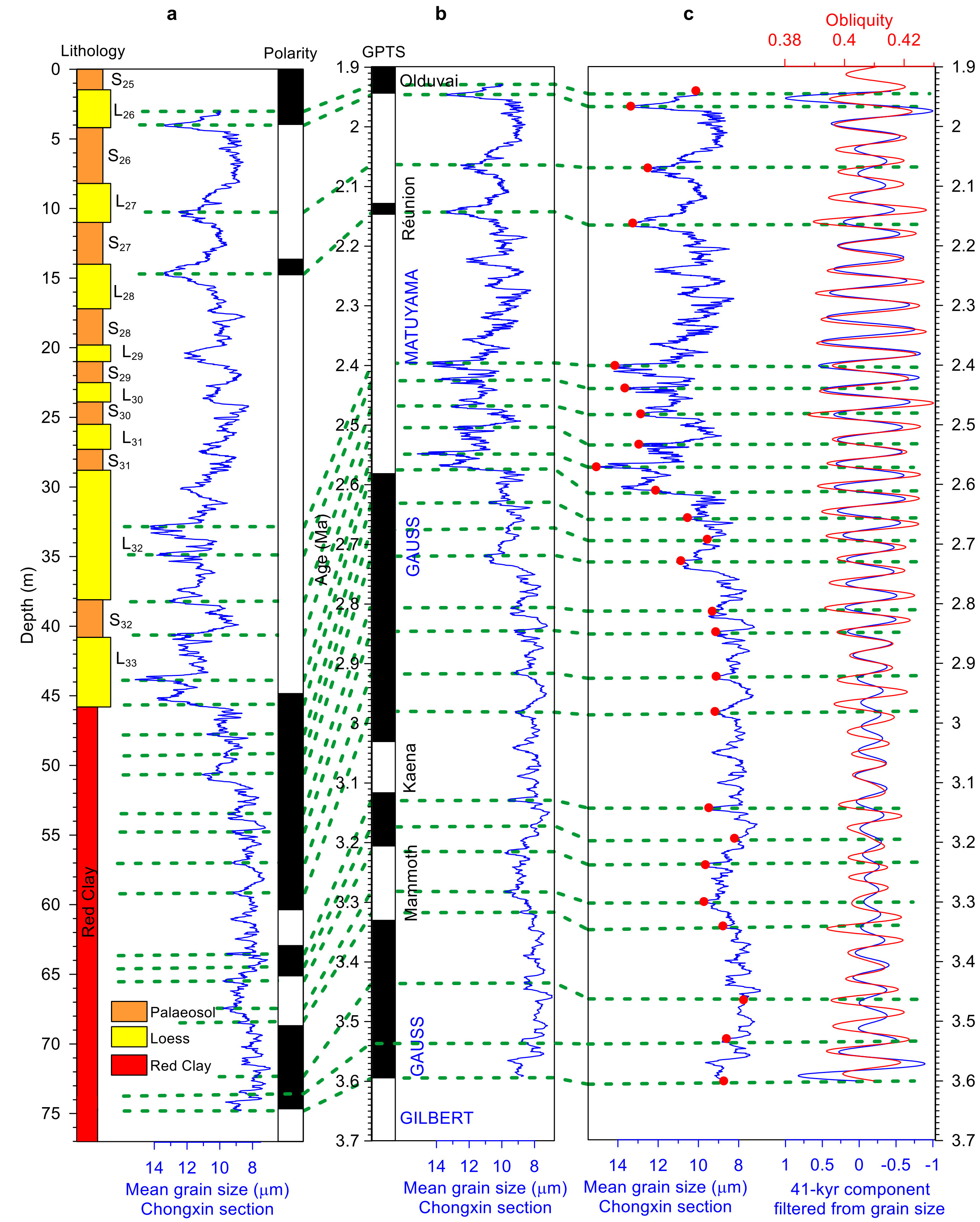

Supplementary Fig. 8. Construction of a refined age model for the Chongxin section by orbital tuning. (**a**) Lithology, mean grain size (seven-point running average of original data), and geomagnetic polarity zones plotted against depth. (**b**) Geomagnetic polarity timescale (GPTS)[15] and mean grain size record plotted with the magnetochronology with respect to age. (**c**) Mean grain size record with tuning points (red dots) and comparison of the 41-kyr component filtered from the tuned grain size record (blue curve) with orbital obliquity[18] (red curve).

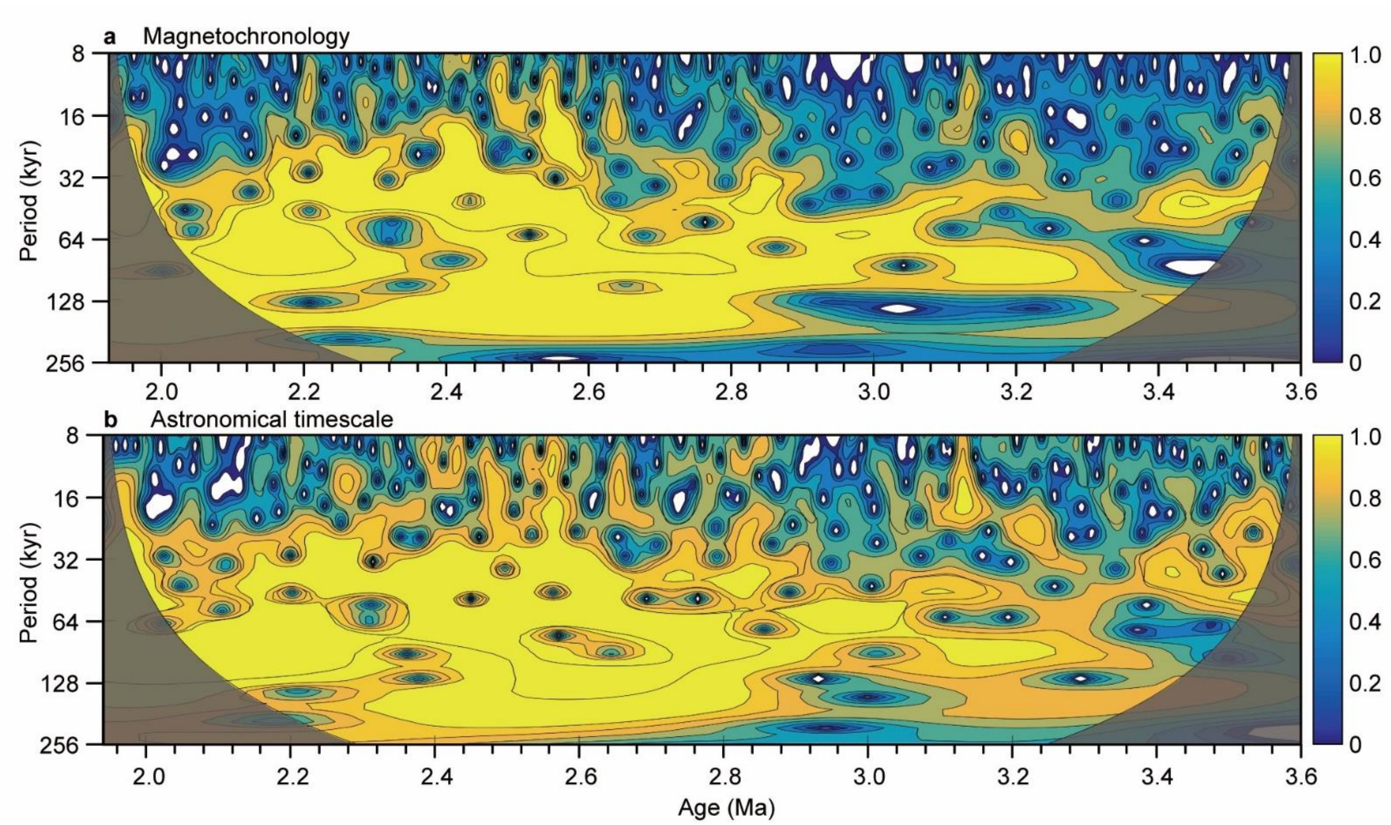

Supplementary Fig. 9. Orbital expression of the Chongxin mean grain size record on the first order magnetochronology and refined astronomical timescale. Wavelet power spectral evolution for the Chongxin mean grain size record on (**a**) the magnetochronology and (**b**) astronomical timescale.

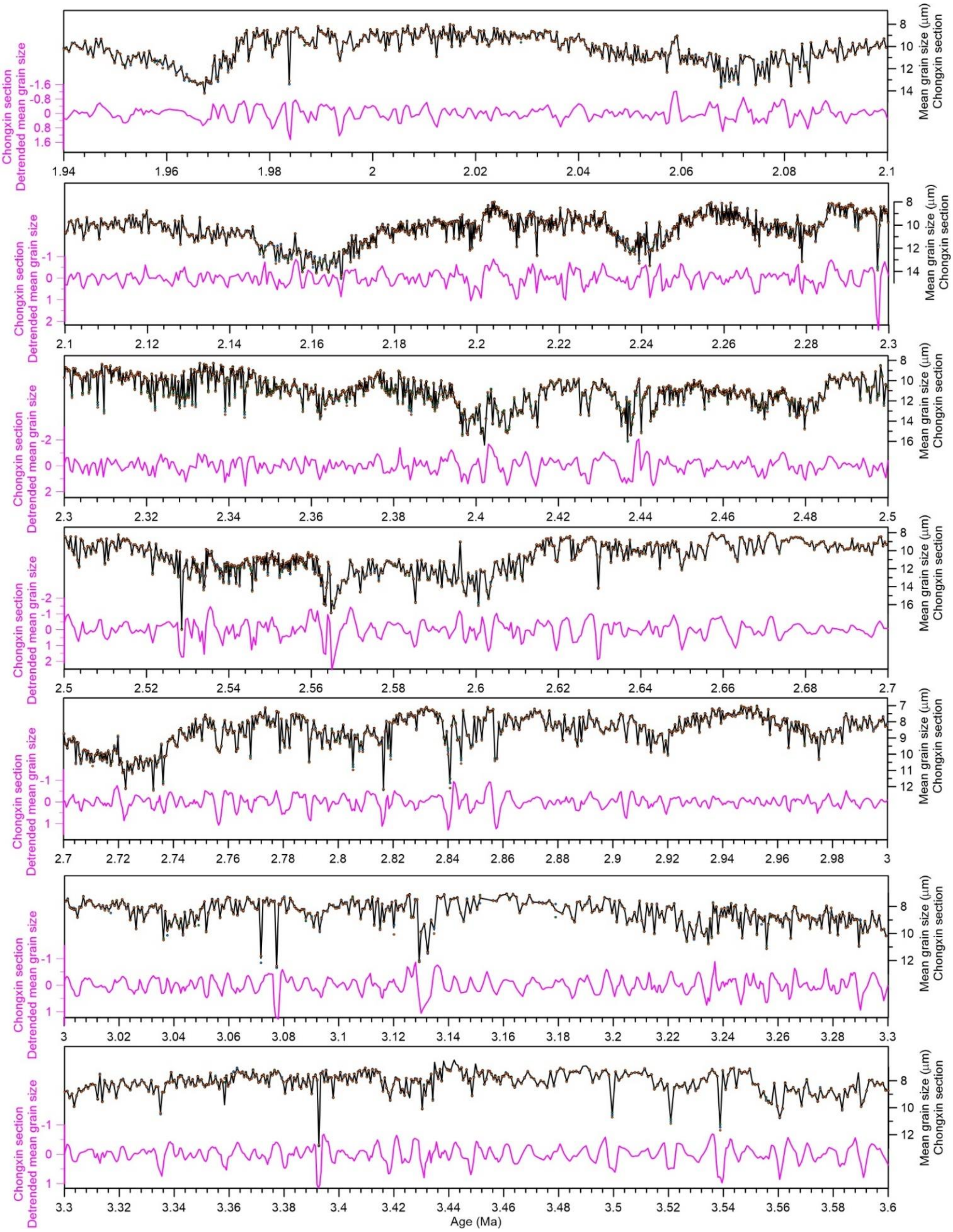

Supplementary Fig. 10. Amplified view of millennial mean grain size variations. Zoomed in view of original (black curve) and < 10 kyr filtered (magenta curve) Chongxin mean grain size time series. We measured the grain size distribution for each sample three times. The original MGS record is obtained from an average of three measurements. Based on the MGS of three measurements, grain size measurement uncertainties are much smaller than the millennial- and orbital-scale variability.

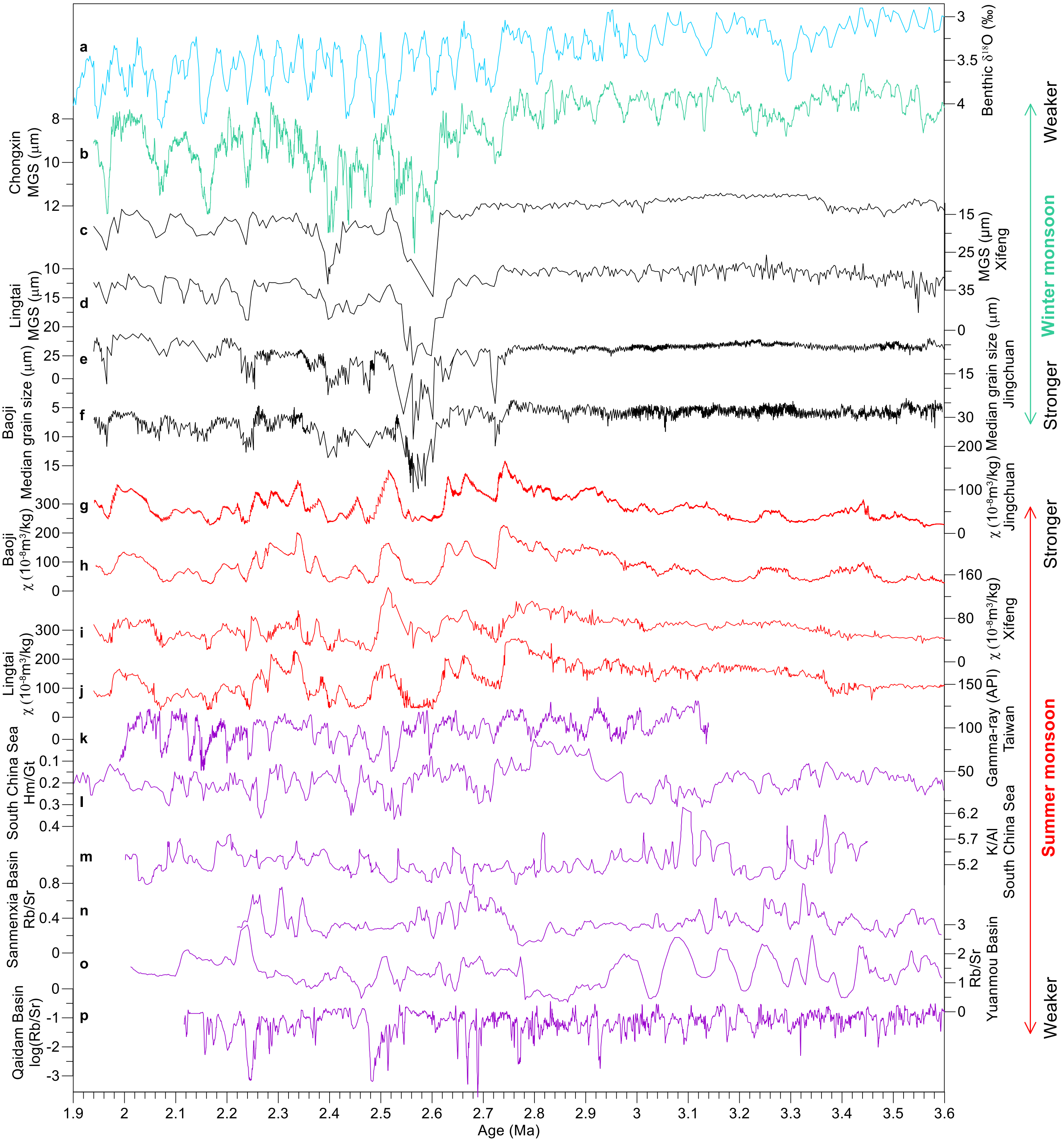

Supplementary Fig. 11. Compiled records of orbital Asian winter and summer monsoon variations across the iNHG. (**a**) LR04 benthic foraminiferal $\delta^{18}O$ record[19]. Mean grain size (MGS) record from the (**b**) Chongxin, (**c**) Xifeng[5], and (**d**) Lingtai[5] sections, median grain size records from the (**e**) Jingchuan[4] and (**f**) Baoji[4] sections, and magnetic susceptibility (χ) records from (**g**) Jingchuan, (**h**) Baoji, (**i**) Xifeng, and (**j**) Lingtai sections[4,5] on our established astronomical age model for the late Pliocene–early Pleistocene loess-palaeosol/red clay sequence. (**k**) Gamma-ray record from Taiwan[8]. (**l**) Hematite to goethite ratio (Hm/Gt) record from the South China Sea[9]. (**m**) K/Al record from the South China Sea[10]. Rb/Sr records of fluvial-lacustrine sediments from the (**n**) Sanmenxia Basin[11], (**o**) Yuanmou Basin[12], and (**p**) Qaidam Basin[13].

Supplementary Table 1. Estimated ages for palaeomagnetic reversal boundaries in the Chongxin astronomical timescale and geomagnetic polarity timescale (GPTS)[15].

Magnetic reversal boundaries	Depth (m)	GPTS (Ma)	Chongxin astronomical timescale	
			Age (Ma)	Difference (Ma)
Bottom of Olduvai	4.0	1.934	1.910	-0.024
Top of Réunion	13.6	2.116	2.059	-0.057
Bottom of Réunion	14.8	2.140	2.078	-0.062
Matuyama–Gauss	44.8	2.595	2.595	0
Top of Kaena	60.4	3.032	3.004	-0.028
Bottom of Kaena	62.9	3.116	3.100	-0.016
Top of Mammoth	65.2	3.207	3.230	0.023
Bottom of Mammoth	68.7	3.330	3.352	0.022
Gauss–Gilbert	74.7	3.596	3.591	-0.005

Supplementary Table 2. Age correlation points used to derive the Chongxin astronomical timescale.

Depth (m)	Age (Ma)
3.00	1.940
4.04	1.967
10.18	2.068
14.80	2.167
32.68	2.398
34.52	2.435
38.04	2.480
40.50	2.531
43.60	2.565
45.38	2.609
47.52	2.650
48.94	2.690
50.40	2.722
53.58	2.816
54.50	2.840
56.84	2.920
58.94	2.975
63.30	3.130
64.22	3.185
65.30	3.234
67.20	3.290
68.26	3.335
72.24	3.464
73.22	3.522
74.80	3.600

Supplementary References

1 Ao, H. *et al.* Late Miocene–Pliocene Asian monsoon intensification linked to Antarctic ice-sheet growth. *Earth Planet. Sci. Lett.* **444,** 75–87 (2016).

2 An, Z. S., Kutzbach, J. E., Prell, W. L. & Porter, S. C. Evolution of Asian monsoons and phased uplift of the Himalaya-Tibetan plateau since Late Miocene times. *Nature* **411,** 62–66 (2001).

3 Sun, Y. B., An, Z. S., Clemens, S. C., Bloemendal, J. & Vandenberghe, J. Seven million years of wind and precipitation variability on the Chinese Loess Plateau. *Earth Planet. Sci. Lett.* **297,** 525–535 (2010).

4 Yang, S. L. & Ding, Z. L. Drastic climatic shift at ~2.8 Ma as recorded in eolian deposits of China and its implications for redefining the Pliocene-Pleistocene boundary. *Quat. Int.* **219,** 37–44 (2010).

5 Sun, Y. B., Clemens, S. C., An, Z. S. & Yu, Z. W. Astronomical timescale and palaeoclimatic implication of stacked 3.6-Myr monsoon records from the Chinese Loess Plateau. *Quat. Sci. Rev.* **25,** 33–48 (2006).

6 Guo, B. *et al.* Dominant precessional forcing of the East Asian summer monsoon since 260 ka. *Geology* **50,** 1372–1376 (2022).

7 Ao, H. *et al.* Global warming-induced Asian hydrological climate transition across the Miocene–

Pliocene boundary. *Nat. Commun.* **12,** 6935 (2021).
8 Vaucher, R. *et al.* Hydroclimate dynamics during the Plio-Pleistocene transition in the northwest Pacific realm. *Glob. Planet. Change* **223,** 104088 (2023).
9 Ao, H., Dekkers, M. J., Qin, L. & Xiao, G. Q. An updated astronomical timescale for the Plio-Pleistocene deposits from South China Sea and new insights into Asian monsoon evolution. *Quat. Sci. Rev.* **30,** 1560–1575 (2011).
10 Tian, J., Xie, X., Ma, W. T., Jin, H. Y. & Wang, P. X. X-ray fluorescence core scanning records of chemical weathering and monsoon evolution over the past 5 Myr in the southern South China Sea. *Paleoceanography* **26,** PA4202 (2011).
11 Zhang, Z. *et al.* East Asian monsoonal climate sensitivity changed in the late Pliocene in response to Northern Hemisphere glaciations. *Geophys. Res. Lett.* **49,** e2022GL101280 (2022).
12 Zhang, Z. *et al.* Low-latitude forcing and high-latitude response of the South Asian summer monsoon through the Pliocene. *J. Geophys. Res.* **128,** e2023JD039057 (2023).
13 Kaboth-Bahr, S. *et al.* A late Pliocene to early Pleistocene (3.3–2.1 Ma) orbital chronology for the Qaidam Basin paleolake (NE Tibetan Plateau) based on the SG-1b drillcore record. *Newsl. Stratigr.* **53,** 479–496 (2020).
14 Zijderveld, J. D. A. in *Methods in Paleomagnetism* (eds D.W. Collinson, K.M. Creer, & S.K. Runcorn) 254–286 (Elsevier, Amsterdam, 1967).
15 Gradstein, F. M., Ogg, J. G., Schmitz, M. D. & Ogg, G. M. *Geologic Time Scale 2020*. (Elsevier, Amsterdam, 2020).
16 Ding, Z. L. *et al.* Stacked 2.6-Ma grain size record from the Chinese loess based on five sections and correlation with the deep-sea $\delta^{18}O$ record. *Paleoceanography* **17,** 1033 (2002).
17 Qiang, X. K., Li, Z. X., Powell, C. M. & Zheng, H. B. Magnetostratigraphic record of the Late Miocene onset of the East Asian monsoon, and Pliocene uplift of northern Tibet. *Earth Planet. Sci. Lett.* **187,** 83–93 (2001).
18 Laskar, J., Fienga, A., Gastineau, M. & Manche, H. La2010: a new orbital solution for the long-term motion of the Earth. *Astron. Astrophys.* **532,** A89 (2011).
19 Lisiecki, L. E. & Raymo, M. E. A Pliocene-Pleistocene stack of 57 globally distributed benthic $\delta^{18}O$ records. *Paleoceanography* **20,** PA1003 (2005).
