# Supplementary material for: Orbital- and millennial-scale Asian winter monsoon variability across the Pliocene–Pleistocene glacial intensification
Source: Nat Commun. 2024 Apr 19;15:3364. doi: 10.1038/s41467-024-47274-9 (PMC11031568; doi:10.1038/s41467-024-47274-9)
Supplement: Supplementary file 3 — Reporting Summary [file 41467_2024_47274_MOESM3_ESM.pdf]

nature portfolio

Corresponding author(s): Hong Ao
Last updated by author(s): Mar 9, 2024

Reporting Summary

Nature Portfolio wishes to improve the reproducibility of the work that we publish. This form provides structure for consistency and transparency in reporting. For further information on Nature Portfolio policies, see our Editorial Policies and the Editorial Policy Checklist.

Statistics

For all statistical analyses, confirm that the following items are present in the figure legend, table legend, main text, or Methods section.

n/a	Confirmed	
☒	☐	The exact sample size (*n*) for each experimental group/condition, given as a discrete number and unit of measurement
☐	☒	A statement on whether measurements were taken from distinct samples or whether the same sample was measured repeatedly
☒	☐	The statistical test(s) used AND whether they are one- or two-sided<br>*Only common tests should be described solely by name; describe more complex techniques in the Methods section.*
☐	☒	A description of all covariates tested
☒	☐	A description of any assumptions or corrections, such as tests of normality and adjustment for multiple comparisons
☒	☐	A full description of the statistical parameters including central tendency (e.g. means) or other basic estimates (e.g. regression coefficient) AND variation (e.g. standard deviation) or associated estimates of uncertainty (e.g. confidence intervals)
☒	☐	For null hypothesis testing, the test statistic (e.g. *F*, *t*, *r*) with confidence intervals, effect sizes, degrees of freedom and *P* value noted<br>*Give P values as exact values whenever suitable.*
☒	☐	For Bayesian analysis, information on the choice of priors and Markov chain Monte Carlo settings
☐	☒	For hierarchical and complex designs, identification of the appropriate level for tests and full reporting of outcomes
☒	☐	Estimates of effect sizes (e.g. Cohen's *d*, Pearson's *r*), indicating how they were calculated

Our web collection on statistics for biologists contains articles on many of the points above.

Software and code

Policy information about availability of computer code

Data collection	No software was used.
Data analysis	We used Grapher 21, ArcGIS (version 10.7), and Adobe illustrator 2020 to drawings figures. We used acycle software 2.6 (MatLab version) to analysis wavelet transform and spectral power.

For manuscripts utilizing custom algorithms or software that are central to the research but not yet described in published literature, software must be made available to editors and reviewers. We strongly encourage code deposition in a community repository (e.g. GitHub). See the Nature Portfolio guidelines for submitting code & software for further information.

Data

Policy information about availability of data

All manuscripts must include a data availability statement. This statement should provide the following information, where applicable:

- Accession codes, unique identifiers, or web links for publicly available datasets
- A description of any restrictions on data availability
- For clinical datasets or third party data, please ensure that the statement adheres to our policy

http://paleodata.ieecas.cn/FrmDataInfo_EN.aspx?id=81837a9f-33af-4feb-916f-c9e09c4a9f53

Research involving human participants, their data, or biological material

Policy information about studies with human participants or human data. See also policy information about sex, gender (identity/presentation), and sexual orientation and race, ethnicity and racism.

Reporting on sex and gender	Our research does not involve human participants, their data, or biological material.
Reporting on race, ethnicity, or other socially relevant groupings	Our research does not involve human participants, their data, or biological material.
Population characteristics	Our research does not involve human participants, their data, or biological material.
Recruitment	Our research does not involve human participants, their data, or biological material.
Ethics oversight	Our research does not involve human participants, their data, or biological material.

Note that full information on the approval of the study protocol must also be provided in the manuscript.

Field-specific reporting

Please select the one below that is the best fit for your research. If you are not sure, read the appropriate sections before making your selection.

☐ Life sciences ☐ Behavioural & social sciences ☒ Ecological, evolutionary & environmental sciences

For a reference copy of the document with all sections, see nature.com/documents/nr-reporting-summary-flat.pdf

Ecological, evolutionary & environmental sciences study design

All studies must disclose on these points even when the disclosure is negative.

Study description	We present a centennial-resolution grain size record between 3.6 and 1.9 Ma from the Chinese Loess Plateau to assess (i) secular evolution, (ii) orbital- and millennial-scale variability, and (iii) the underlying dynamics of the AWM across the iNHG, which is marked by substantial changes of climate-cryosphere boundary conditions.
Research sample	Our study site, the Chongxin section (35°15′N, 107°8′E), is located on the central Chinese Loess Plateau under the influence of seasonally alternating Asian summer monsoon and winter monsoon circulation. We sampled the loess-palaeosol/red clay sequence from the Chongxin section for Asian winter monsoon research.
Sampling strategy	After removal of surface outcrop, we collected 3,571 unoriented samples and 251 oriented block samples from the Chongxin section.
Data collection	Our grain size data analyses for each sample were measured by using a Malvern 3000 Laser Instrument with a Hydro LV wet dispersion unit from 0.02 µm to 2000 µm
Timing and spatial scale	Data collection: October 2021-February 2022.
Data exclusions	No data were excluded from the analyses.
Reproducibility	Each sample's grain size distribution was measured three times. The attempts to repeat the experiment were successful.
Randomization	Samples were allocated according to their depths.
Blinding	Very low.

Did the study involve field work? ☒ Yes ☐ No

Field work, collection and transport

Field conditions	We collected samples in summer in Chongxin, where the temperature was around 37 ? and the annual precipitation was around 80 mm.
Location	The Chongxin section (35°15′N, 107°8′E) at the Gansu province, China. It is located on the central Chinese Loess Plateau.
Access & import/export	We have an introduction letter from the Institute of Earth Environment, Chinese Academy of Sciences, Xi'an. We informed the local government before sampling.

Disturbance	No significant disturbance.

Reporting for specific materials, systems and methods

We require information from authors about some types of materials, experimental systems and methods used in many studies. Here, indicate whether each material, system or method listed is relevant to your study. If you are not sure if a list item applies to your research, read the appropriate section before selecting a response.

Materials & experimental systems

n/a	Involved in the study
☒	☐ Antibodies
☒	☐ Eukaryotic cell lines
☒	☐ Palaeontology and archaeology
☒	☐ Animals and other organisms
☒	☐ Clinical data
☒	☐ Dual use research of concern
☒	☐ Plants

Methods

n/a	Involved in the study
☒	☐ ChIP-seq
☒	☐ Flow cytometry
☒	☐ MRI-based neuroimaging

Plants

Seed stocks	No seed stocks were used.
Novel plant genotypes	No novel plant genotypes.
Authentication	No authentication.
